# Selective Photocatalytic Oxidation of Glycerol and 3-Pyridinemethanol by Nanotube/Nanowire-Structured TiO_2_ Powders Obtained by Breakdown Anodization

**DOI:** 10.3389/fchem.2022.856947

**Published:** 2022-05-12

**Authors:** Sıdıka Çetinkaya, Gofur Khamidov, Levent Özcan, Leonardo Palmisano, Sedat Yurdakal

**Affiliations:** ^1^ Kimya Bölümü, Fen-Edebiyat Fakültesi, Afyon Kocatepe Üniversitesi, Ahmet Necdet Sezer Kampüsü, Afyonkarahisar, Turkey; ^2^ Biyomedikal Mühendisliği Bölümü, Mühendislik Fakültesi, Afyon Kocatepe Üniversitesi, Ahmet Necdet Sezer Kampüsü, Afyonkarahisar, Turkey; ^3^ Schiavello-Grillone Photocatalysis Group, Università degli Studi di Palermo, Dipartimento di Ingegneria (DI), Palermo, Italy

**Keywords:** glycerol, 3-pyridinemethanol, nanotube structured TiO_2_, heterogeneous photocatalysis, selective oxidation, vitamin B_3_, green synthesis

## Abstract

Nanotube/nanowire-structured TiO_2_ was formed on the Ti surface by an anodic oxidation method performed at different potential values (50 or 60 V) and for different times (3 or 5 h). The TiO_2_ photocatalysts were taken in powder form using the ultrasonic treatment from the Ti electrodes, calcined at different temperatures, and characterized by XRD and SEM techniques, and BET surface area analyses. Both the crystallinity and the size of the primary TiO_2_ particles increased by increasing the heat treatment temperature. While all the photocatalysts heat treated up to 500°C were only in the anatase phase, the particles heat-treated at 700°C consisted of both anatase and rutile phases. The BET specific surface area of the samples decreased drastically after heat treatment of 700°C because of partial sinterization. SEM analyses indicated that the prepared materials were structured in both nanotubes and nanowires. They were tested as photocatalysts for the selective oxidation of glycerol and 3-pyridinemethanol under UVA irradiation in water at room temperature and ambient pressure. Glyceraldehyde, 1,3-dihydroxyacetone, and formic acid were determined as products in glycerol oxidation, while the products of 3-pyridinemethanol oxidation were 3-pyridinemethanal and vitamin B_3_. Non-nanotube/nanowire-structured commercial (Degussa P25 and Merck TiO_2_) photocatalysts were used for the sake of comparison. Low selectivity values towards the products obtained by partial oxidation were determined for glycerol. On the contrary, higher selectivity values towards the products were obtained (total 3-pyridinemethanal and vitamin B_3_ selectivity up to ca. 90%) for the photocatalytic oxidation of 3-pyridinemethanol. TiO_2_ photocatalysts must be highly crystalline (calcined at 700°C) for effective oxidation of glycerol, while for the selective oxidation of 3-pyridinemethanol it was not necessary to obtain a high crystallinity, and the optimal heat treatment temperature was 250°C. Glycerol and its oxidation products could more easily desorb from highly crystalline and less hydroxylated surfaces, which would justifies their higher activity. The prepared photocatalysts showed lower activity than Degussa P25, but a greater selectivity towards the products found.

## Introduction

Biodiesel is produced by the acid- or base-catalyzed transesterification of triglycerides. In biodiesel production, approximately 110 kg of crude glycerol is formed as an intermediate product from 1 ton of biodiesel (Behr et al., 2008). However, as biodiesel production increases, so does the production of its main by-product, glycerol (1,2,3-propantriol) (Yang et al., 2012).

The significant increase in biodiesel production in recent years has led to a large surplus of glycerol in the market. Therefore, it is essential to develop active catalysts to convert glycerol into various high added value products (Dodekatos et al., 2018a; Dodekatos et al., 2018b). According to the statistical estimates made by the United Nations Food and Agriculture Organization (OECD-FAO), until 2025, biodiesel production will continue to increase, and this means that there will be always an excess of glycerol in the coming years (Dodekatos et al., 2018a). Furthermore, due to its low price and low demand, glycerol is sometimes disposed of as waste, thus, posing a serious threat to the environment (Gholami et al., 2014).

Selective photocatalytic oxidation reactions that are conducted under environmentally friendly conditions and with acceptable costs, appear promising to produce valuable chemicals for use in the chemical and pharmaceutical industries (Colmenares and Luque 2014). Some research groups have published studies on the oxidation of glycerol with photocatalytic methods (Augugliaro et al., 2010; Maldotti and Molinari 2011; Dodekatos et al., 2018a). Compounds of commercial importance can be obtained due to partial oxidation of glycerol (Dodekatos et al., 2018a; Dodekatos et al., 2018b). 1,3-Dihydroxyacetone is one of them and it is used mainly as a tanning agent in the cosmetic industry (Criminna et al., 2006; Behr et al., 2008; Bagheri et al., 2015) and as starting material for the synthesis of many compounds in organic chemistry. Another important molecule is glyceraldehyde (GAD), an intermediate of the carbohydrate metabolism and a standard for chiral molecules (D- or L-) (Behr et al., 2008). Formic acid is also obtained from glycerol and is used in the leather industry in Asia, in agriculture in Europe (Dodekatos et al., 2018a) and its salts in fuel cells.

Photocatalytic oxidation of 3-pyridinemethanol was also carried out in this work. Vitamin B_3_ (pyridine-3-carboxylic acid), one of its main oxidation products, is actively used to prevent alcoholism and pellagra disease, and it is produced in high amounts worldwide (Alfe et al., 2014). Only a few research papers have been published on the partial photocatalytic oxidation of 3-pyridinemethanol. All these studies have been carried out in water without the addition of organic solvents. Some authors (Alfe et al., 2014; Spasiano et al., 2015a, 2015b, 2016) carried out this reaction under acidic conditions (pH 1–4) and using commercial TiO_2_ and TiO_2_-graphene composite photocatalysts. These reactions took place in the presence of Cu^2+^ in the absence of oxygen. By increasing pH from 1 to 4, both aldehyde and vitamin B_3_ yields and Cu^2+^ ions conversion decreased, and the reactions almost did not occur at pH higher than 4, under the experimental conditions used (Alfe et al., 2014; Spasiano et al., 2015a, 2015b, 2016).

Yurdakal et al. (2017) investigated the selective oxidation of pyridinemethanols (*o-, m-, p-*) under UVA, UV-Vis, and visible light sources with Pt-loaded TiO_2_ photocatalysts at pH’s 2–12. High selectivity towards vitamin B_3_ was obtained in a basic medium using low crystalline (mainly amorphous) rutile TiO_2_ samples prepared at room temperature. The mainly amorphous TiO_2_ photocatalysts which have hydrophilic surface, allows the desorption of the products before their further oxidation up to their complete mineralization. This behavior is opposite to that of glycerol (Augugliaro et al., 2010) and its partial oxidation products which, having more hydrophilic OH groups, desorb with difficulty from a highly hydroxylated surface (Augugliaro et al., 2008).

Another recent photocatalytic oxidation study on 3-pyridinemethanol was carried out by Çetinkaya and Yurdakal (2021). Home-prepared TiO_2_ photocatalysts were prepared from TiCl_4_ precursor at room temperature, 60 and 100°C in the presence of HCl, HNO_3_, or H_2_SO_4_.

The industrial synthesis of vitamin B_3_ is performed at high temperature and pressure by oxidizing picolinic isomers with nitric acid, permanganate or chromic acid in the presence of vanadia-titania-zirconia oxide supported catalysts (Yurdakal et al., 2017).

The present study describes the formation of nanotube-structured TiO_2_ samples on the metal Ti surface by the anodic oxidation method by applying different voltage values for various times. These materials were removed in the form of powder from the surface of the Ti plate by ultrasonic method, subjected to heat treatments at different temperatures and characterized. The photocatalysts were tested for the selective photocatalytic oxidation of glycerol and 3-pyridinemethanol in water and under UVA irradiation. TiO_2_-structured nanotubes on the Ti plates are interesting because of their high surface area (i.e., ca. 90 m^2^/g in this work) with respect to thin films, strong ion-exchange ability, effective photocatalytic activities, and easy production by electrochemical oxidation methods (Cui et al., 2009; Roy et al., 2011; Fraoucene et al., 2019). The anodic oxidation technique is one of the most used techniques due to its easy application and low cost (Huang et al., 2016). To obtain metal oxide tube arrays or pore arrays on the metal surface it is essential to carry out the anodization process in the presence of a suitable electrolyte medium (Roy et al., 2011). Anodization parameters (such as applied potential, electrolyte composition/concentration and time) can be easily changed to control some morphologic parameters (Gong et al., 2001).

## Experimental Section

### Preparation of Ti Plates

Ti plates of 1 mm thickness were cut by guillotine to be 5.0 cm × 8.0 cm. To smooth the surface of the plates, they were sanded with 800, 1,000, 1,200, and 1,500 grit sandpapers, respectively. Then, they were cleaned in an ultrasonic bath for 10 min in acetone, ethanol and distilled water, respectively.

The Ti plates were then chemically cleaned in a solution medium containing 4% HF, 31% HNO_3_, and 65% water for 30 s (Özcan et al., 2018). Afterward, the Ti plates were again cleaned in an ultrasonic bath in water for 10 min and dried at room temperature to remove all contaminating ions coming from the use of HF and HNO_3_.

### Preparation of TiO_2_ Photocatalysts

The two-electrode system in which the anodic oxidation process is applied to form nano-structured TiO_2_ samples on the Ti plate surface is shown in Supplementary Figure S1. The solution in which anodic oxidation will be carried out was prepared by dissolving 0.3% by mass of NH_4_F in a solution containing 2% water and 98% ethylene glycol by volume (Özcan et al., 2018). Anodic oxidation was carried out in this prepared electrolyte solution by applying a definite constant voltage (50 or 60 V) to the electrodes for a certain time (3 or 5 h) and washed with pure water. These chosen values are similar to those used in the literature (Özcan et al., 2018). Then, the anode was placed in a beaker containing pure water and subjected to an ultrasonic bath, which allowed TiO_2_ in nanotube/nanowire structure to break and separate from the plate (Ali and Hannula 2017; Ali et al., 2018; Yang et al., 2019).

A sufficient amount of TiO_2_ nanoparticles were obtained by applying this process repeatedly. The crystallinity of the obtained TiO_2_ nanoparticles was very low. In order to obtain photocatalysts of different crystallinity, the partitioned TiO_2_ photocatalyst was kept in an air atmosphere for 3 h (Protherm, PLF-110/10 model) (temperature rise rate: 3°C/min) in a muffle furnace at different temperatures (100–700°C).

The resulting photocatalysts were named TiO_2_-xV-yh-z. Here, x: voltage value applied while preparing photocatalyst by anodic oxidation, y: time (h) during which the voltage was applied, and z: thermal treatment temperature of the anode. For example, the powdered photocatalyst TiO_2_-60V-3h-250 was prepared at 60 V for 3 h, separated from the titanium plate by means of an ultrasonic bath, and calcined at 250°C. The photoanode used without thermal treatment for the sake of comparison was named TiO_2_-xV-yh-25 because it was prepared at room temperature (ca. 25°C).

The potential value applied in the production of all nano-structured electrodes produced by anodic oxidation was reached by increasing ∼0.2 V per second from a voltage of 0.0 V.

### Characterization Experiments

The produced nano-structured TiO_2_ samples were characterized by X-ray diffraction (XRD) which was performed with a Bruker D8 Advance diffractometer using the Cu Kα radiation (1.544 Å) and a 2θ scan rate of 1.281°/min, while scanning electron microscope observations (SEM) were performed with an FEI microscope (NanoSEM 650 model, FEI Company). The samples on the stab were covered with a thin gold film before SEM observations. XRD analyses were performed to establish the crystal phase(s) and the primary particle sizes of the photocatalysts, while SEM analyses to determine their morphological properties.

BET specific surface area values of the prepared samples were determined by the multi-point BET method using a Micromeritics (Gemini VII model) apparatus. The nano-structured TiO_2_ photocatalysts were degassed at 200°C for 5 h before the measurement. The crystalline and morphologic properties of the samples could be changed only slightly by the used degassing temperature, however we could not perform degassing process at low temperatures because the pores would not be completely free and the measurement would be wrong (i.e., room temperature).

### Photocatalytic Test System

A 250 mL Pyrex beaker was used as a photoreactor for experiments performed under UVA light (Supplementary Figure S2). UVA rays reaching the suspension were provided by four fluorescent lamps (Philips, each lamp is 8 W) emitting mainly at 365 nm. The spectrum of the lamp is shown in Supplementary Figure S3. The distance to the suspension of these lamps was 6.8 cm, and the lamps were placed parallel to each other. The average value of the radiation energy reaching the suspensions, determined using a radiometer (Delta Ohm, DO9721), was 21 W m^−2^ in the range 315–400 nm.

The initial substrate concentration for glycerol was 2 or 10 mM while for 3-pyridinemethanol was 0.50 mM. The amount of used photocatalyst was 0.20 g L^−1^. Before the lamps were turned on, the suspension was stirred in the dark for 30 min to reach thermodynamic and adsorption-desorption equilibrium. Moreover, the suspension was continuously stirred magnetically at 500 rpm during the photocatalytic experiments that were carried out at pH 7 and at room temperature (ca. 25°C). The aqueous suspension was in contact with the atmosphere which provided the necessary oxygen. Samples withdrawn at certain time intervals were filtered through a hydrophilic membrane (0.45 µm, HA, Millipore) before being injected in the high performance liquid chromatography (HPLC) and total organic carbon (TOC) analyzers. All the photocatalytic experiments were carried out twice or three times to check their reproducibility.

### Analytical Techniques

Qualitative and quantitative analyses of the samples withdrawn from the experimental system at certain times during the photocatalytic experiments of glycerol and 3-pyridinemethanol were performed with HPLC (Shimadzu Prominence LC-20A model and SPD-M20A photodiode array detector). Two consecutive C-18 columns were used (Phenomenex and Syncronis, respectively), and the column temperature was kept at 30°C. Retention times of analyzed substrates and intermediates and UVA spectra of compounds were compared with known standards (Sigma-Aldrich, purity ≥ 98%). For glycerol and its intermediates, the mobile phase was a 5 mM aqueous solution of sulfuric acid, and the flow rate was 0.3 cm^3^ min^−1^. For 3-pyridinemethanol and its intermediates, the mobile phase was 40% methanol and 60% deionized water, and the flow rate was 0.2 cm^3^ min^−1^.

Total organic carbon (TOC) analyses were performed with a Shimadzu (TOC-LCPN model) device to determine the amount of mineralized carbon dioxide during the oxidation of the substrates studied. The selectivity towards the main products (%) and the substrate conversion (%) have been calculated according to the formula below:

$$\text{\%}\;\text{Selectivity}=\frac{\left(\text{Amount}\;\text{of}\;\text{product}\;\text{formed},\text{mmol}\right)}{\left(\text{Amount}\;\text{of}\;\text{substrate}\;\text{reacted},\text{mmol}\right)}\text{x}100\text{\%}$$

$$\text{\%}\;\text{Conversion}=\frac{\left(\text{Amount}\;\text{of}\;\text{reacted}\;\text{substrate},\text{mmol}\right)}{\left(\text{Initial}\;\text{amount}\;\text{of}\;\text{substrate},\;\text{mmol}\right)}\text{x}100\text{\%}$$

## Results and Discussion

### Characterization of Photocatalysts

**FIGURE 1:**
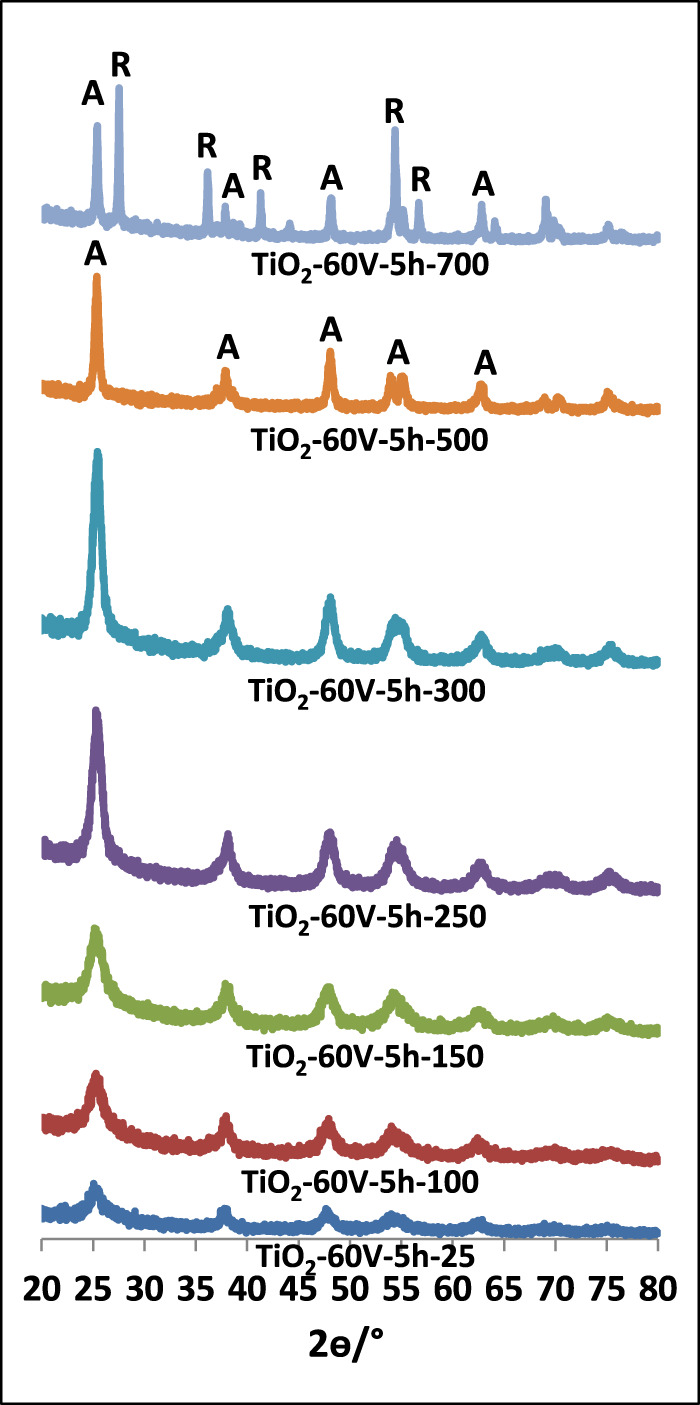
XRD patterns of TiO_2_-60V-5h samples calcined at different temperatures.

**FIGURE 2:**
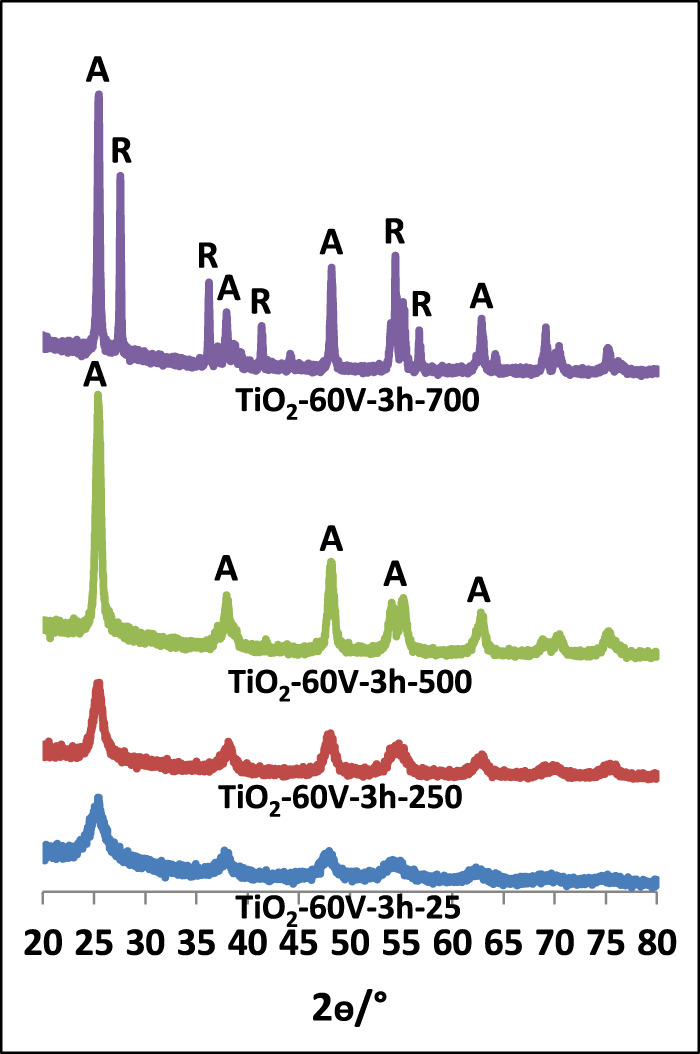
XRD patterns of TiO_2_-60V-3h samples calcined at different temperatures.

**FIGURE 3:**
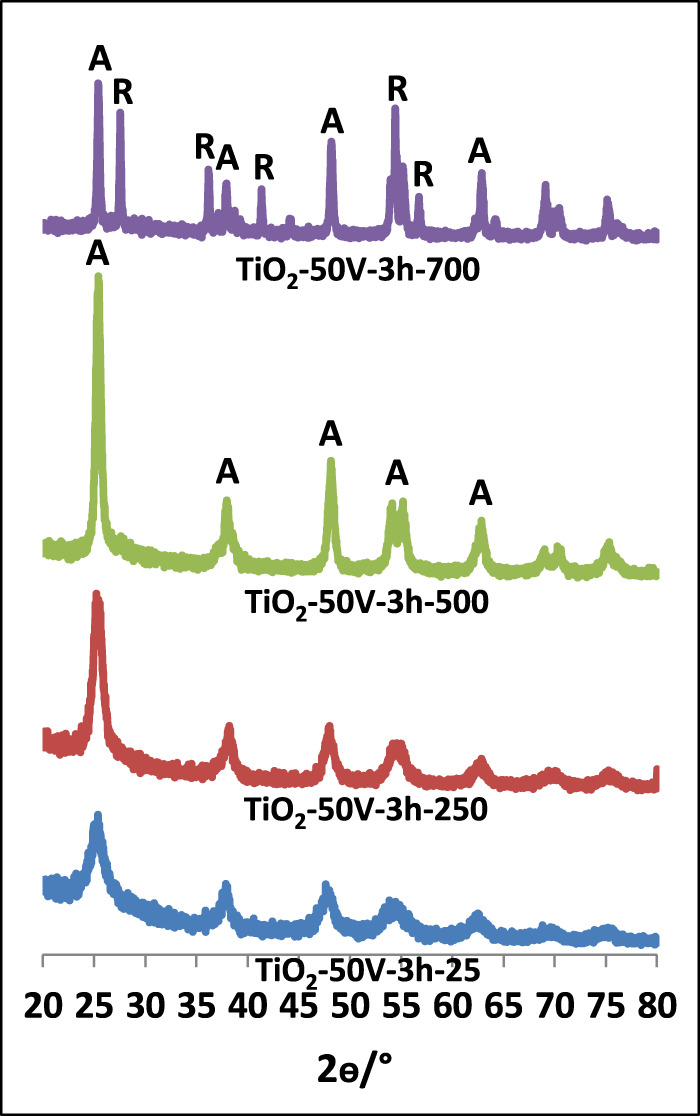
XRD patterns of TiO_2_-50V-3h samples calcined at different temperatures.

Figures 1–3 show XRD patterns of nanotube/nanowire-structured TiO_2_ photocatalysts calcined at different temperatures (up to 700°C). Since the behaviors of all three sample types (TiO_2_-60V-5h, TiO_2_-60V-3h, and TiO_2_-50V-3h) with respect to heat treatment were similar, TiO_2_-60V-5h was calcined at several different temperatures, while TiO_2_-60V-3h and TiO_2_-50V-3h samples were calcined only at 250, 500, and 700°C. The peak values ​​at 2θ = 27.5°, 36.5°, 41.0°, 54.1°, and 56.5° refer to the rutile phase, while those at 2θ = 25.58°, 38.08°, 48.08°, 54.58° belong to the anatase phase (Yurdakal et al., 2012; Fiorenza et al., 2020). Low and broad XRD peaks are indicative of a low crystallinity, consequently, uncalcined TiO_2_ samples showed to have a low crystallinity (high amorphous content) and a small amount of anatase phase content (Yurdakal et al., 2012; Çetinkaya and Yurdakal 2021). For all samples, as the calcination temperature increased up to 500°C, the intensity and the sharpness of the anatase phase peak increased also. Consequently, TiO_2_ crystallinity increased by increasing the thermal treatment temperature. At 700°C, a sharp peak belonging to the rutile phase was also formed. The XRD peak intensity of the anatase phase decreased at 700°C with respect to the sample calcined at 500°C, because the transformation from anatase phases to rutile occurred.

Supplementary Figure S4 shows the XRD patterns of commercial TiO_2_ (Merck and Degussa P25) photocatalysts. These photocatalysts, which are not in nanotube/nanowire structure, are in powder form and have been used to compare the investigated home prepared photocatalysts. Merck TiO_2_ photocatalyst is entirely in the anatase phase, while Degussa P25 is in the anatase and rutile phases (ca. 82% A, 18% R).

In Table 1, phases of TiO_2_ samples, percentage of each crystal phase, and primary particle size values ​​of each phase calculated from the Scherrer equation are reported. As the calcination temperature of the photocatalysts raised, the primary particle sizes increased due to the sintering of the particles. When the TiO_2_-60V-5h samples were examined, although the primary particle size raised slightly up to 300°C, it showed a significant increase at 500°C and especially at 700°C. The primary particle size value was only 10 nm at 300°C, while it increased at 18 and 34 nm at 500 and 700°C, respectively. Both anatase and rutile phases were formed for all samples calcined at 700°C. Anatase and rutile percentages of these samples are different. The rutile phase was dominant in TiO_2_-60V-5h-700, while anatase phase was present in other samples (especially TiO_2_-60V-3h-700). The primary particle size of the anatase phase for all samples was about 33 nm, while that of the rutile phase 52 nm. In Degussa P25, the most common commercial photocatalyst, the primary particle size values ​​of both phases are close to each other and are approximately 20 nm.

**TABLE 1:** Crystal phases, phase percentages and primary particle sizes of photocatalysts.

Photocatalyst	Crystal phase	Primary particle size of anatase phase (nm) for 2θ = 25°	Primary particle size of rutile phase (nm) for 2θ = 27.5°
TiO_2_-60V-5h-25	A	7.6	50
TiO_2_-60V-5h-100	A	6.6	
TiO_2_-60V-5h-150	A	6.4	
TiO_2_-60V-5h-250	A	8.8	
TiO_2_-60V-5h-300	A	10	
TiO_2_-60V-5h-500	A	18	
TiO_2_-60V-5h-700	A (41%) R (59%)	34	
TiO_2_-60V-3h-25	A	6.7	
TiO_2_-60V-3h-250	A	9.8	
TiO_2_-60V-3h-500	A	16	
TiO_2_-60V-3h-700	A (83%) R (17%)	33	56
TiO_2_-50V-3h-25	A	7.4	
TiO_2_-50V-3h-250	A	9.0	
TiO_2_-50V-3h-500	A	16	
TiO_2_-50V-3h-700	A (53%) R (47%)	32	52
Degussa P25	A (82%) R (18%)	20	18
Merck	A	39	

**FIGURE 4:**
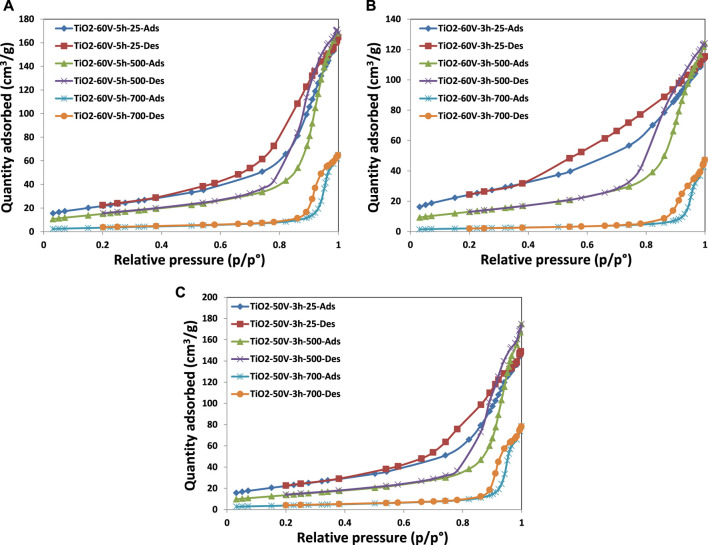
BET isotherms of the TiO_2_ samples: **(A)** TiO_2_-60V-5h, **(B)** TiO_2_-60V-3h, and **(C)** TiO_2_-50V-3h.

Figure 4 shows the nitrogen adsorption-desorption isotherms of the prepared TiO_2_ samples, and the results derived from these isotherms are reported in Table 2. All the prepared TiO_2_ photocatalysts showed the type IV isotherm characteristic with an H3-type loop according to the IUPAC classification, indicating the irregular structure of the inner surface of the pores (Sing et al., 1985; Yurdakal et al., 2019; Zhang et al., 2022). The samples prepared without thermal treatment showed values of specific surface area in the range ca. 78–89 m^2^/g; however, their surface area decreased to ca. 47–54 m^2^/g after calcination at 500°C. A drastic reduction of the surface area up to 7.4 m^2^/g occurred after the treatment at 700°C. This finding is due to the sinterization of the nanotubes at a very high temperature (see SEM results). The BET isotherms of the three different samples (TiO_2_-60V-5h, TiO_2_-60V-3h and TiO_2_-50V-3h) showed the same behavior if the thermal treatment temperature was the same, 25, 500, or 700°C (see Figure 4).

**TABLE 2:** BET specific surface area, pore volume and pore width values of the TiO_2_ samples.

Photocatalyst	BET surface area (m^2^/g)	BJH adsorption cumulative volume of pores between 1.7 and 300 nm width (cm³/g)	BJH adsorption average pore width (nm)
^a^TiO_2_-60V-5h-25	78	0.25	11
TiO_2_-60V-5h-500	54	0.26	16
TiO_2_-60V-5h-700	12	0.096	26
^a^TiO_2_-60V-3h-25	89	0.17	7.0
TiO_2_-60V-3h-500	47	0.19	13
TiO_2_-60V-3h-700	7.4	0.066	31
^a^TiO_2_-50V-3h-25	80	0.22	9.7
TiO_2_-50V-3h-500	49	0.26	18
TiO_2_-50V-3h-700	13	0.11	28
Degussa P25	64	0.20	10
Merck	11	0.030	20

a The properties of these samples could be slightly changed after degassing process that was performed at 200°C.

**FIGURE 5:**
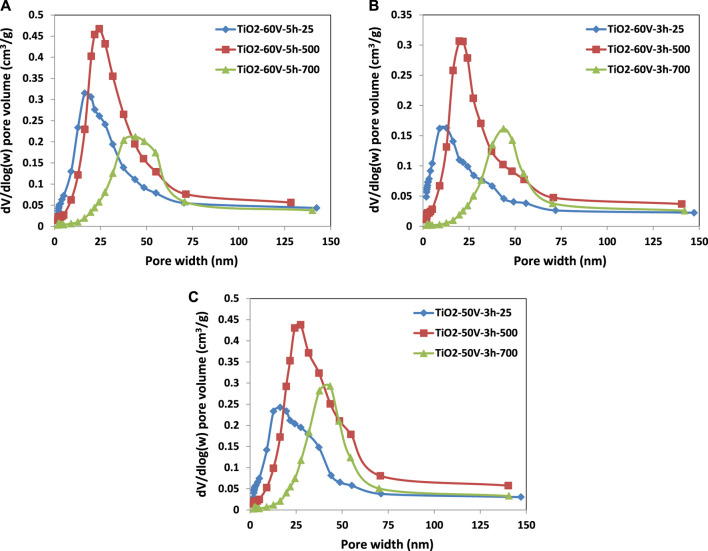
Pore size distribution versus pore volume of the TiO_2_ samples: **(A)** TiO_2_-60V-5h, **(B)** TiO_2_-60V-3h, and **(C)** TiO_2_-50V-3h.

**FIGURE 6:**
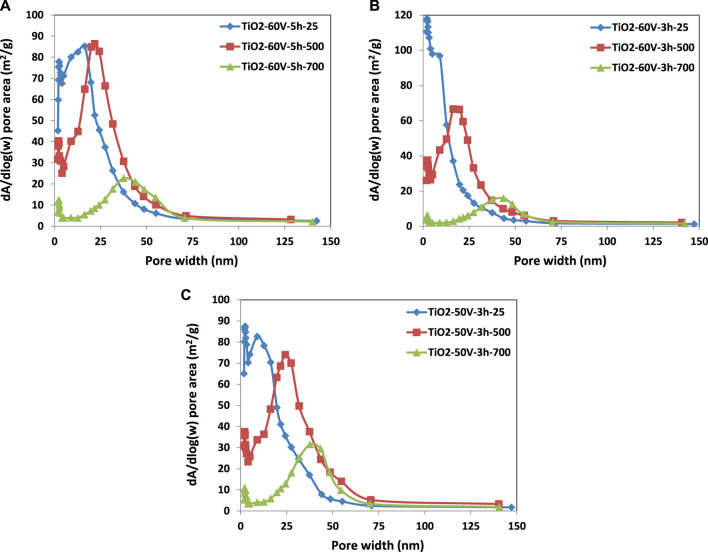
Pore size distribution versus pore area of the TiO_2_ samples: **(A)** TiO_2_-60V-5h, **(B)** TiO_2_-60V-3h, and **(C)** TiO_2_-50V-3h.

BJH Adsorption cumulative volume values (between 1.7 and 300 nm width) of pores of the samples, treated thermally at room temperature and 500°C, are similar. In contrast, that of the treated ones at 700°C decreased significantly. For instance, the pore volume of TiO_2_-60V-5h-500 was 0.261 cm³/g, while TiO_2_-60V-5h-700 was only 0.0957 cm³/g because of the sinterization of the TiO_2_ nanotubes. Indeed, the BET adsorption-desorption isotherm of TiO_2_-60V-5h-700 is very different from those of TiO_2_-60V-5h-25 and TiO_2_-60V-5h-500; it has a tiny hysteresis loop compared with the other two. In addition, BJH adsorption mean pore width values of the samples increased with the heat treatment temperature as there is an opposite trend between pore volume and width. Consequently, as seen in Figures 5, 6, pore size distribution curves were shifted to larger values by increasing the temperature of thermal treatment. For instance, peak maximum of the pore width vs. pore volume curves of TiO_2_-60V-3h-25, TiO_2_-60V-3h-500, and TiO_2_-60V-3h-700 are ca. 13, 22, and 44 nm, respectively (see Figure 6B).

In this section, also some SEM images of nanotube/nanowire-structured TiO_2_ samples prepared using the anodic oxidation method on the Ti surface at different potential values and different times are discussed. In order to obtain a sufficient amount of photocatalyst, TiO_2_ structures were formed on the Ti surface many times by the anodic oxidation method and removed from the surface by ultrasonic treatment.

**FIGURE 7:**
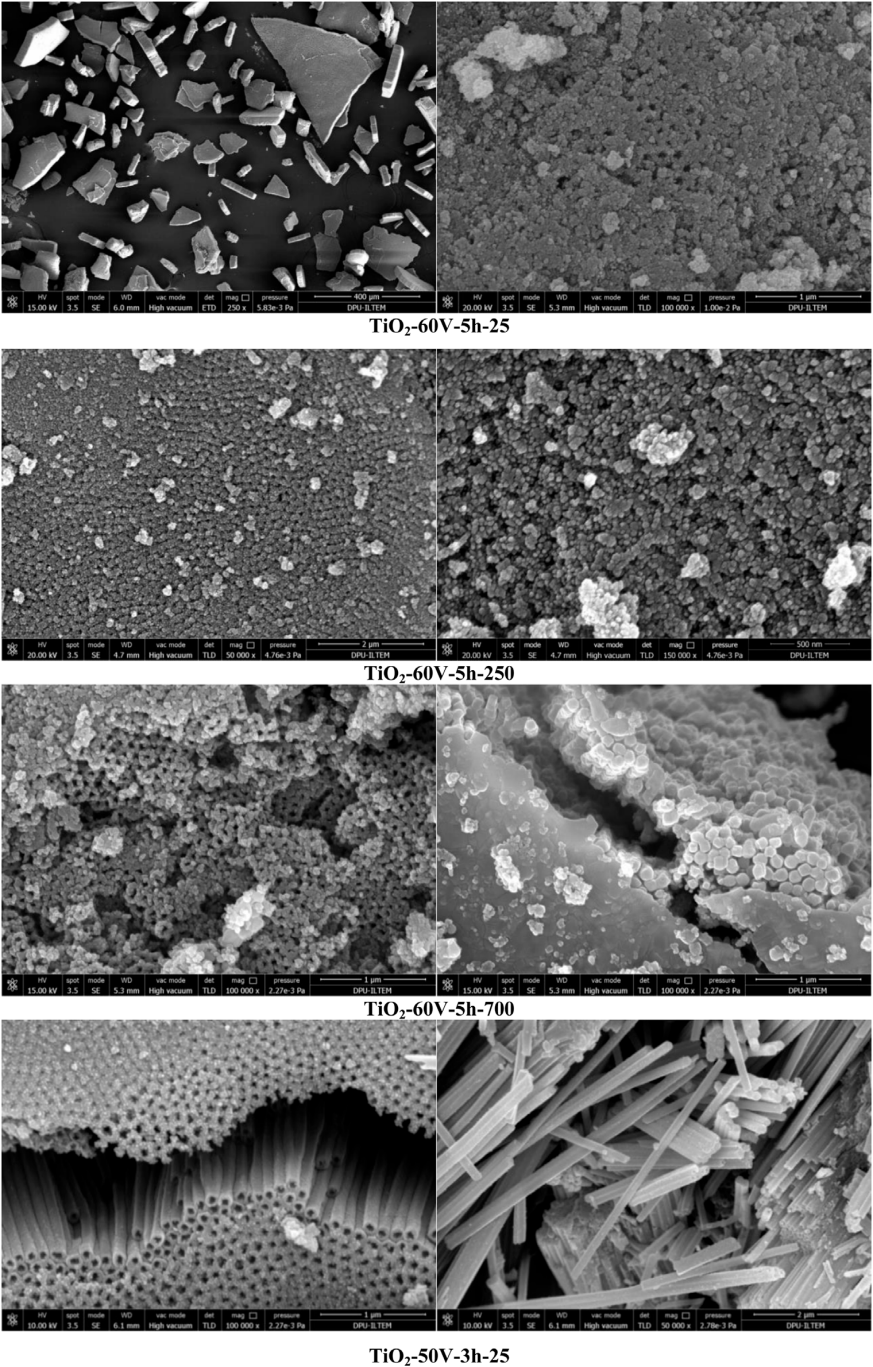
SEM images of the prepared photocatalysts.

Figure 7; Supplementary Figure S5 show SEM images of TiO_2_-60V-5h-25 nanoparticles taken at different magnifications. The TiO_2_ particles show a heterogeneous distribution of their size from a few microns to 400 μm, but the size of most of them ranges between 50 and 100 µm. Figure 7 reports SEM images taken from the top of the nanomaterial. These pictures show that the structure is porous and in a nanotube structure. However, there are also nanotube structures with closed mouths on the surface. Supplementary Figures S5B–D are cross-sectional views of TiO_2_-60V-5h-25 material. From these images, the diameter of the TiO_2_ nanotube is between 100 and 150 nm, and according to the picture in Supplementary Figure S5C, the nanotube length is about 8 µm.

In Figure 7 the SEM images of the same material (TiO_2_-60V-5h) calcined in the air at 250°C (TiO_2_-60V-5h-250) can be observed. Figure 7 is top images of the material, with a clear nanotube structure. Supplementary Figures S6A,B are filled nanotube/nanowire photos. In the picture of Supplementary Figure S6B, it can be noticed that some nanowire structures are separated from each other. The diameters of these nanowires are between about 60 and 90 nm. These SEM images indicate that some nanowire structures formed together with the nanotube structure. The separation of some of the nanowire structures from each other also increases the effective surface area of the material. Supplementary Figure S6C is a cross-sectional view of this material, which shows that the section thickness of the nanostructure is approximately 18 µm.

SEM images of TiO_2_-60V-5h-700 are provided in Figure 7. Figure 7; Supplementary Figure S7A are images of the upper part of the material that appears to be in nanotube structures. Due to the high calcination temperature of 700°C, its crystallinity is quite high and some sintering can be observed in Figure 7. Indeed, the BET surface area of this sample was very low with respect to the uncalcined ones because of the partial sintering of the nanostructures; 12.2 vs. 78.3 m^2^/g. Supplementary Figure S7B is a side sectional view of the same material.

SEM images of the TiO_2_-50V-3h-25 nanomaterial are given in Figure 7. Supplementary Figure S8A shows the overall particle distribution. This photocatalyst has smaller particles (up to about 100 µm) than the “TiO_2_-60V-3h” materials. Figure 7; Supplementary Figure S8B show the nanotube structures in the cross-sectional view. These photos were taken from the upper part of the material, and nanotube structures with a homogeneous distribution are evident. Supplementary Figure S8D reports an image of the bottom of the material. According to the cross-sectional view, the length of the nanotubes is approximately 9 µm (Supplementary Figure S8E). In some places, the nanotubes were separated from the main structure, individually or combined (Figure 7). The diameter of these nanotubes is approximately 140–160 nm.

SEM photos of TiO_2_-60V-3h-25 sample are given in Supplementary Figure S9. Large nanoparticles up to about 180 µm are present (Supplementary Figure S9A). There are also smaller particles of the order of nanometers in size. Supplementary Figure S9B indicates that the material is mostly in nanowire structure. The diameter of the nanostructures is about 100–135 nm (Supplementary Figures S9E,F). Supplementary Figure S9C shows the traces left by the broken nanowires on the surface. According to Supplementary Figure S9D, the layer length of the nanomaterial is approximately 8 µm. Supplementary Figure S10 gives an SEM picture of the same material calcined at 500°C. The nanostructures in Supplementary Figure S10B appear to consist of tiny crystals with a 20–30 nm diameter.

### Photocatalytic Activity

Photocatalytic glycerol oxidation experiments were carried out under UVA irradiation at pH 7 to work under environmentally friendly conditions (Yurdakal et al., 2017). No activity was detected in the presence of light but in the absence of photocatalyst or in the dark in the presence of photocatalyst (Augugliaro et al., 2012). Light, photocatalyst and oxygen were essential for the occurrence of the photocatalytic reaction.

**SCHEME 1:**
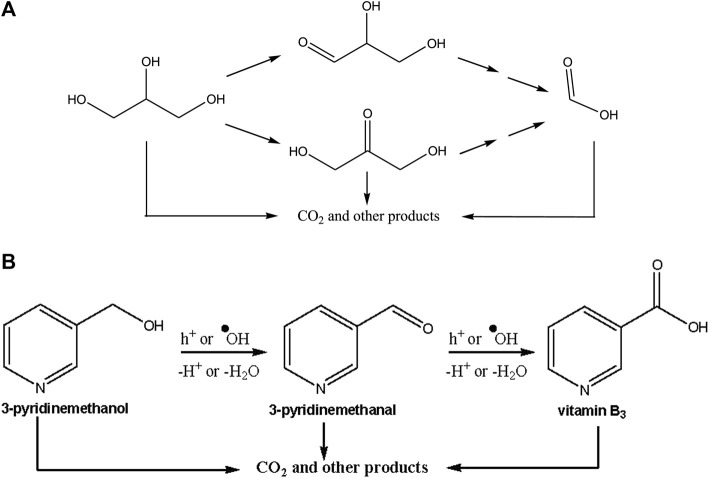
The proposed mechanism for photocatalytic oxidations of glycerol **(A)** and 3-pyridinemethanol **(B)** to their photocatalytic oxidation products (Yurdakal et al., 2020).

Table 3 shows the results of photocatalytic experiments performed with 10 mM initial concentration of glycerol. The conversion values ​​are low for our prepared samples due to the high initial concentration. From the qualitative and quantitative analyses of the samples during the reaction, glyceraldehyde (GAD), 1,3-dihydroxyacetone (DHA), and formic acid (FA) were determined as the main intermediates (see Scheme 1A). The low carbon balance is due to the formation of undetected aliphatic species. We could not detect the other intermediates, because of their trace amounts. In addition, CO_2_ was formed as a mineralization product. In the tables of the photocatalytic experiments conversion, selectivity (*vs*. the formation of GAD, DHA and FA), TOC decrease (%), and the pH values ​​of the suspension at the end of the experiment after 8 h are reported. TOC analyses were carried out only for some experiments. In general, the pH value decreased due to the formation of FA and other acidic species. While the photocatalysts prepared on the Ti plate surface showed low glycerol conversion (up to 10%), the commercial Degussa P25 photocatalyst gave rise to a 27% conversion that was the highest value among the photocatalysts tested, and consequently the highest TOC decrease was achieved with this photocatalyst. First of all, the experiments were carried out with the TiO_2_-60V-5h photocatalyst at different temperatures. TiO_2_-60V-5h-500 photocatalyst gave the highest conversion with 9%. Therefore, other photocatalysts were calcined at 500°C (TiO_2_-50V-3h-500 and TiO_2_-60V-3h-500) and were also used for comparison purposes. Among them, TiO_2_-50V-3h-500 showed 10% conversion. This photocatalyst is in the anatase crystalline phase. Photocatalysts with low heat treatment showed to have scarce activities. Since the selectivity values were calculated at different reaction times, there is an inverse relationship between the selectivity values and the conversion values. In fact, as the reaction progresses, the intermediate products formed are more likely to reoxidize several times.

**TABLE 3:** The results of photocatalytic glycerol (10 mM) oxidation under UVA light with TiO_2_ photocatalysts.

Photocatalyst	X_8h_ (%)	^a^S_GAD_ (%), X_8h_	^b^S_DHA_ (%), X_8h_	^c^S_FA_ (%), X_8h_	−∆TOC (%)	^d^pH X_8h_
No photocatalyst	0	—	—	—	0	7.0
Degussa P25	27	3	3	5	45	4.0
Merck	20	28	7	6	38	4.0
TiO_2_-60V-5h-25	4	38	13	17	traces	3.9
TiO_2_-60V-5h-100	6	24	9	10	traces	4.0
TiO_2_-60V-5h-150	7	25	9	8	traces	3.9
TiO_2_-60V-5h-200	3	56	17	20	traces	4.2
TiO_2_-60V-5h-250	2	52	17	25	traces	5.0
TiO_2_-60V-5h-300	5	25	9	15	3	5.8
TiO_2_-60V-5h-500	9	13	7	7	1	6.6
TiO_2_-60V-5h-700	7	22	10	10	2	5.0
TiO_2_-50V-3h-500	10	6	6	5	1	7.0
TiO_2_-60V-3h-500	8	9	5	6	1	4.7

^a^S_GAD_, ^b^S_DHA_, and ^c^S_FA_: selectivities towards glyceraldehyde, 1,3-dihydroxyacetone and formic acid for conversion after 8 h of reaction (X_8h_), respectively. X_8h_: Conversion values for a reaction time of 8 h. ^d^pH: pH value measured after 8 h of reaction (X_8h_).

Therefore, the TiO_2_-60V-5h-200 photocatalyst with the lowest activity showed the highest GAD and DHA selectivity, i.e., 56% and 17%, respectively. Although the photocatalysts have a porous structure, as they detached from the Ti surface in large pieces (in microns), their effective surface is less than those in powder and showed a lower activity than the commercial ones. Furthermore, the selectivity values towards GAD are higher than those towards DHA. This is due to the fact that to obtain DHA it is necessary to oxidize the carbon in position two of glycerol, while for the formation of GAD it is necessary to oxidize one of the carbons in position 1 or 3. Consequently, the probability of obtaining GAD is greater (Augugliaro et al., 2010).

Since the conversion observed during photocatalytic experiments carried out with an initial concentration of 10 mM glycerol was low, a set of photocatalytic experiments were also performed starting from an initial concentration of 2 mM glycerol (see Table 4). Due to the low initial glycerol concentration, the amount of GAD in some experiments was too low to be analysed, and it was not reported in table. As the calcination temperature of each type of nanomaterial increased, the glycerol conversion values increased, and the highest conversions were obtained with samples calcined at 700°C. The most significant result was found for the TiO_2_-50V-3h photocatalyst, with glycerol conversions of 5, 14 and 25% for TiO_2_-50V-3h-25, TiO_2_-50V-3h-500 and TiO_2_-50V-3h-700, respectively. In particular, the TiO_2_-50V-3h-700 sample proved to be much more photoactive per surface unit than the corresponding samples treated at lower temperatures. This finding indicates that crystallinity, higher at higher temperatures, is the most important factor for the photocatalytic oxidation of glycerol. Glycerol, a highly hydrophilic molecule, could interact very strongly with the surface of uncalcined TiO_2_ samples which are mainly in the amorphous phase (Augugliaro et al., 2008). Furthermore, its oxidation products (i.e., DHA and GAD) could remain on the surface blocking the active sites for a longer time than what happens on hydrophobic surfaces. Conversely, glycerol and its oxidation products could more easily desorb from crystalline and probably less hydroxylated surfaces, which would justify greater activity of the latter (Augugliaro et al., 2010). For this reason the Merck TiO_2_ sample was shown to possess the highest activity despite its specific surface area being quite small (approx. 11 m^2^/g).

**TABLE 4:** The results of photocatalytic glycerol (2 mM) oxidation under UVA light with TiO_2_ photocatalysts.

Photocatalyst	X_8h_ (%)	^a^S_GAD_, (%) X_8h_	^b^S_DHA_, (%) X_8h_	^c^S_FA_, (%) X_8h_	−∆TOC (%)	^d^pH X_8h_
No photocatalyst	—				—	
Degussa P25	47	56	3	3	19	7.8
Merck	53		9	12	5	4.7
TiO_2_-50V-3h-25	5		19	35	traces	3.7
TiO_2_-50V-3h-500	14		8	11	2	6.0
TiO_2_-50V-3h-700	25		6	5	4	7.8
TiO_2_-60V-3h-25	13	18	6	14	traces	3.9
TiO_2_-60V-3h-500	11		8	10	2	5.6
TiO_2_-60V-5h-25	13	26	10	12	traces	4.3
TiO_2_-60V-5h-500	15		6	9	2	4.6
TiO_2_-60V-5h-700	17	14	10	9	3	5.7

^a^S_GAD_, ^b^S_DHA_, and ^c^S_FA_: selectivities towards glyceraldehyde, 1,3-dihydroxyacetone and formic acid for conversion after 8 h of reaction (X_8h_), respectively. X_8h_: Conversion values for a reaction time of 8 h ^d^pH: pH value measured after 8 h of reaction (X_8h_).

Some works were published on the partial photocatalytic oxidation of glycerol (Augugliaro et al., 2010; Imbault et al., 2020; Payormhorm and Idem 2020; Rangarajan et al., 2021). Previously, we performed photocatalytic oxidation of glycerol in aqueous suspensions containing home-prepared TiO_2_ photocatalysts (Augugliaro et al., 2010). The initial glycerol concentration (50–180 mM) and the reaction time were higher than in the present work. Moreover, the photocatalysts were bulk anatase, rutile, or anatase–rutile polymorphic phases. In that work, Merck and Degussa P25 TiO_2_ photocatalysts showed higher activity/selectivity than the home-prepared ones, like in the present study. The fact that a high crystallinity of the photocatalysts is beneficial for the activity is confirmed by the present investigation. In addition, we also detected the same intermediates. Consequently, the TiO_2_ morphology, whether nanotube/nanowire or bulk structure, does not play a significant role in the product nature and distribution.

Payormhorm and Idem (2020) used C-doped TiO_2_ prepared by a sol-microwave method for photocatalytic glycerol oxidation under visible light irradiation. Mainly DHA, GAD, and FA products and trace amounts of acetic acid were obtained. Unlike Payormhorm and Idem (2020), in our work, we used UVA irradiation and did not detect acetic acid.

Imbault et al. (2020) investigated photocatalytic oxidation of glycerol to DHA, GAD, glyceric acid, and several other chemicals using TiO_2_ photocatalysts under simulated solar irradiation and in acetonitrile as solvent. Reaction rate and selectivity towards the products were higher in acetonitrile compared with an aqueous suspension. For instance, after 6 h of reaction, glycerol conversion and selectivity towards DHA were 96.8% and 17.8% in acetonitrile compared to 36.1% and 14.7% in water, respectively.

Rangarajan et al. (2021) prepared Ag-AgBr/TiO_2_ ternary nanocomposites for selective photocatalytic glycerol oxidation into GAD and DHA in a non-aqueous solvent under visible light and aerobic conditions. Although a lower glycerol conversion was obtained in acetonitrile than in water after 4 h, a significantly higher yield of DHA (13%) and GAD (19%) was obtained due to the less significant formation of non-selective ˙OH radicals in the organic solvent.

Oxidation of 3-pyridinemethanol, an aromatic primary alcohol, was also carried out with the same photocatalysts used for glycerol oxidation. 3-Pyridinemethanal and vitamin B_3_ were determined as main products. Scheme 1B shows a proposed mechanism for photocatalytic oxidation starting from 3-pyridinemethanol. The first step can be considered the interaction of h^+^ or hydroxyl radicals with formation of 3-pyridinemethanal subsequently oxidized to vitamin B_3_. The over-oxidation products of these substrates and their corresponding molecules were mainly aliphatic species and CO_2_ (Yurdakal et al., 2020).

Table 5 shows the results of the photocatalytic oxidation tests of 3-pyridinemethanol oxidation under UVA light in the presence of TiO_2_-50V-3h photocatalysts obtained at different thermal treatment temperatures. Initial reaction rates, pseudo-first-order rate constants, substrate conversion values ​​for one and 3 h reaction times, selectivity towards 3-pyridinemethanal and vitamin B_3_ after 15% and 30% conversion, and pH values ​​of the suspension at the end of the reaction are given in the table. Only 15% conversion was achieved after 3 h with the TiO_2_-50V-3h-25 photocatalyst. This photocatalyst appeared to be in the anatase phase, but its crystallinity was low. Although crystalline samples showed higher activity, it can be noted that TiO_2_-50V-3h-250 photocatalyst was both the most active and the most selective towards 3-pyridinemethanal and vitamin B_3_ formation. In fact, the k-value for the 3-pyridinemethanol oxidation of this photocatalyst was about 3 times that of the TiO_2_-50V-3h-25 photocatalyst (0.156 h^−1^ vs. about 0.052 h^−1^). In addition, 87% 3-pyridinemethanal and 11% vitamin B_3_ selectivities were obtained after 30% conversion.

**TABLE 5:** Photocatalytic results of 3-pyridinemethanol (0.5 mM) oxidation under UVA light with TiO_2_-50V-3h, TiO_2_-60V-3h, TiO_2_-60V-5h, and the commercial photocatalysts prepared at different calcination treatment temperatures.

Photocatalyst	-r_0_x10^3^ (mM·h^−1^)	kx10^3^ (h^−1^)	X_1h_ (%)	X_3h_ (%)	^a^S_3-Pyridinemethanal,_ (%)		^b^S_Vitamin B3,_ (%)		^c^pH X_3h_
					X_0.15_	X_0.30_	X_0.15_	X_0.30_	
No photocatalyst	0	0	0	0					7
TiO_2_-50V-3h-25	15	52	3	15	88		11		6.7
TiO_2_-50V-3h-250	91	156	16	38	90	87	7	11	6.7
TiO_2_-50V-3h-500	98	121	15	32	52	53	12	11	7
TiO_2_-50V-3h-700	77	111	12	29	51	52	13	11	6.9
TiO_2_-60V-3h-25	23	45	3	14	89		11		5.7
TiO_2_-60V-3h-250	87	144	15	36	89	85	7	11	6.1
TiO_2_-60V-3h-500	79	110	13	29	60	58	10	10	7
TiO_2_-60V-3h-700	58	100	10	26	63	62	9	9	7
TiO_2_-60V-5h-25	47	107	9	27	72	74	8	9	5.0
TiO_2_-60V-5h-100	31	108	7	27	84	79	11	11	4.5
TiO_2_-60V-5h-150	54	148	11	38	85	82	11	12	4.7
TiO_2_-60V-5h-200	92	273	17	56	81	77	10	12	5.9
TiO_2_-60V-5h-250	124	276	23	57	85	80	11	12	5.5
TiO_2_-60V-5h-300	86	189	16	44	78	71	12	15	5.6
TiO_2_-60V-5h-500	94	139	16	35	53	53	10	12	5.4
TiO_2_-60V-5h-700	75	96	11	26	54	55	10	11	6.3
Merck	54	86	9	23	55	54	8	8	7
Degussa P25	298	596	48	84	40	39	11	11	7

−r_0_: initial reaction rate, k: pseudo-first-order rate constant, ^a^S_3-Pyridinemethanal_ and ^b^S_Vitamin B3_: selectivities towards 3-pyridinemethanal and vitamin B_3_ for 15% (X_0.15_) and 30% (X_0.30_) conversions. X_1h_ and X_3h_: Conversion values after 1 and 3 h of reaction, respectively. ^c^pH: pH value measured after 3 h of reaction (X_3h_).

In this work seems that the temperature of 250°C, at least for the samples prepared as above described, is the optimum choice both for the conversion of the substrates studied and for production of 3-pyridinemethanal and vitamin B_3_ (see Table 5). It should be noted that the significantly higher selectivity values towards 3-pyridinemethanal compared to vitamin B_3_ may be due to the relatively short reaction times and consequently to the low conversion values. In fact, the first oxidation product deriving from 3-pyridinemethanol is aldehyde (3-pyridinemethanal), while acid vitamin B_3_ is subsequently formed. The amount of vitamin B_3_, therefore, increased with the reaction time, while the amount of 3-pyridinemethanal decreased. The carbon balance values were quite good, indicating that the CO_2_ formation resulting from complete mineralization of the substrate was negligible; consequently, TOC values were not inserted in the following tables. Finally, there was no significant change in the pH values measured at the end of the experiment, and this finding confirms that vitamin B_3_ was formed in small quantities.

In Table 5, the results of the photocatalytic oxidation of 3-pyridinemethanol under UVA light with TiO_2_-60V-3h photocatalysts obtained at different thermal treatment temperatures are given. TiO_2_-60V-3h-250 photocatalyst gave the highest activity (k = 0.144 h^−1^) with 36% conversion. This photocatalyst, which is thermally treated at 250°C, still has the highest activity and product selectivity values. It showed 85% 3-pyridinemethanal and 11% vitamin B_3_ selectivity (96% total) for 30% conversion.

Photocatalytic results under UVA light of 3-pyridinemethanol oxidation with TiO_2_-60V-5h photocatalysts calcined at different temperatures are given in Table 5. It can be noted that 250°C was the optimum temperature. The values of the rate constant with respect to the calcination temperature of the photocatalysts can be arranged almost like a Gaussian curve. The optimum calcination temperature depends on the type of substrate. The highly crystalline TiO_2_ samples were found to be the most effective both in terms of conversion and selectivity for the oxidation of glycerol (best results in the presence of calcined TiO_2_ at 700°C), while a medium/low crystallinity of TiO_2_ was required for the oxidation of 3-pyridinemethanol (best results in the presence of calcined TiO_2_ at 250°C).

**FIGURE 8:**
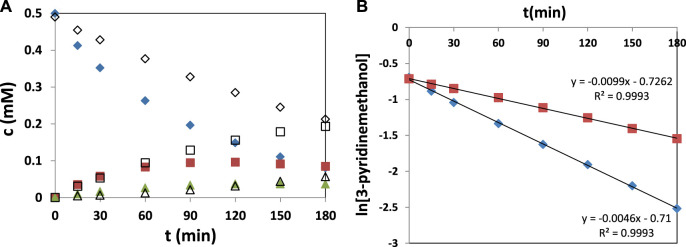
**(A)** The results of photocatalytic oxidation of 3-pyridinemethanol (♦,◊) to 3-pyridinemethanal (■,□) and vitamin B_3_ (▲,Δ) versus time in the presence of Degussa P25 (full symbols) and TiO_2_-60V-5h-250 (empty symbols) under UVA light at pH 7, **(B)** the pseudo-first-order kinetic values of Degussa P25 (♦) and TiO_2_-60V-5h-250 (■).

Table 5 also shows the results of two commercial TiO_2_ photocatalysts (Degussa P25, Merck) for the sake of comparison. The commercial TiO_2_ catalysts were prepared at high temperatures, and their high crystallinity was evidenced by their sharp and intense XRD peaks (Yurdakal et al., 2020). TiO_2_-60V-5h-250 showed the highest activity among the home prepared photocatalysts, and in particular the k-value was about 3 times higher than that found for the Merck commercial photocatalyst (0.276 vs. 0.0856 h^−1^). Conversely, Degussa P25 showed greater photocatalytic activity (k = 0.596 h^−1^). However, while the total selectivity value of aromatic aldehyde and vitamin B_3_ after the 30% substrate conversion was only 50% in the case of Degussa P25, this value was 92% for the TiO_2_-60V-5h-250 sample. The photocatalytic oxidation results of 3-pyridinemethanol versus time in the presence of Degussa P25 and TiO_2_-60V-5h-250 are shown in Figure 8A. It was evidenced in Figure 8B that the reaction rate fits the pseudo-first-order kinetics.

Although the conversion rate of alcohol was lower in the presence of TiO_2_-60V-5h-250 than in Degussa P25, the TiO_2_-60V-5h-250 photocatalyst produced more products. This finding indicates that the latter photocatalyst (TiO_2_-60V-5h-250) can potentially be used for fast and selective photocatalytic synthetic reactions under environmentally friendly conditions, although a handicap is the laborious preparation process.

Some authors (Alfe et al., 2014; Spasiano et al., 2015a, 2015b, 2016) performed this reaction under acidic conditions (pH 1–4) using commercial TiO_2_ and also TiO_2_-graphene composite photocatalysts. The reaction took place in the presence of Cu^2+^ in the absence of oxygen, but at pH >4 the reaction almost did not occur (Alfe et al., 2014; Spasiano et al., 2015a, 2015b, 2016). Our group investigated the selective oxidation of pyridinemethanols (o-, m-, p-) under UVA, UV-Vis, and visible light sources in the presence of Pt-loaded TiO_2_ photocatalysts at pH 2–12 (Yurdakal et al., 2017). High selectivity towards vitamin B_3_ was obtained only in basic conditions. Another recent study on photocatalytic oxidation of 3-pyridinemethanol was carried out by Çetinkaya and Yurdakal (2021). In that case, samples prepared at low temperature showed high product selectivity with respect to Degussa P25. However, in the present work we investigated the thermal treatment temperature in detail and found that the optimum thermal treatment is 250°C. Low crystalline TiO_2_ photocatalysts have a hydrophilic surface, but allowed the desorption of the products before their further oxidation up to a complete mineralization, and this is the reason for the high product selectivity (Augugliaro et al., 2008).

## Conclusion

In this study, nanotube-structured TiO_2_ samples were prepared by anodic oxidation method at different potential values (50 or 60 V) ​​applied for different times (3 or 5 h) on a Ti metal surface. Moreover the obtained samples after they were taken by ultrasonic treatment from the TiO_2_ plate, were subjected to heat treatment at different temperatures. As the temperature of the heat treatment increased, the crystallization of TiO_2_ and the size of the primary particles of the materials increased. All the photocatalysts were found to be only in the anatase phase until the calcination temperature was not greater than 500°C. At 700°C, however, significant quantities of both the anatase and rutile phases were obtained. SEM analyses showed that the prepared materials were in nanotube structure, but also porous structures and nanowires were noted.

The TiO_2_ photocatalysts were tested for photocatalytic selective oxidation of glycerol and 3-pyridinemethanol under UVA light under environmentally friendly conditions. Two non-nanotube/nanowire structured commercial TiO_2_ photocatalysts (Degussa P25 and Merck) were used for the sake of comparison. The home-prepared TiO_2_-60V-5h-250 photocatalyst showed both a high activity and a high selectivity towards the main products of the oxidation of 3-pyridinemethanol (over 90% total selectivity towards 3-pyridinemethanal and vitamin B_3_). For glycerol oxidation the best photocatalysts were those more crystalline calcined at 700°C even if their specific surface areas are the smallest with respect to others.

## Data Availability

The original contributions presented in the study are included in the article/Supplementary Material, further inquiries can be directed to the corresponding author.
