# Supplementary material for: Selective Photocatalytic Oxidation of Glycerol and 3-Pyridinemethanol by Nanotube/Nanowire-Structured TiO2 Powders Obtained by Breakdown Anodization
Source: Front Chem. 2022 May 12;10:856947. doi: 10.3389/fchem.2022.856947 (PMC9135064; doi:10.3389/fchem.2022.856947)
Supplement: Supplementary file 1 [file DataSheet1.docx]

Supporting Information

**Selective photocatalytic oxidation of glycerol and 3-pyridinmethanol by nanotube/nanowire-structured TiO_2_ powders obtained by breakdown anodization**

Sıdıka Çetinkaya^a^, Gofur Khamidov^a^, Levent Özcan^b^, Leonardo Palmisano^c^, Sedat Yurdakal^a,*^

^a^Kimya Bölümü, Fen-Edebiyat Fakültesi, Afyon Kocatepe Üniversitesi, Ahmet Necdet Sezer Kampüsü, 03200 Afyonkarahisar, Turkey.

^b^Biyomedikal Mühendisliği Bölümü, Mühendislik Fakültesi, Afyon Kocatepe Üniversitesi, Ahmet Necdet Sezer Kampüsü, 03200 Afyonkarahisar, Turkey.

^c^Schiavello-Grillone Photocatalysis Group, Università degli Studi di Palermo, Dipartimento di Ingegneria (DI), Viale delle Scienze, 90128 Palermo, Italy.

*Corresponding authors e-mail: [sedatyurdakal@gmail.com](mailto:sedatyurdakal@gmail.com)


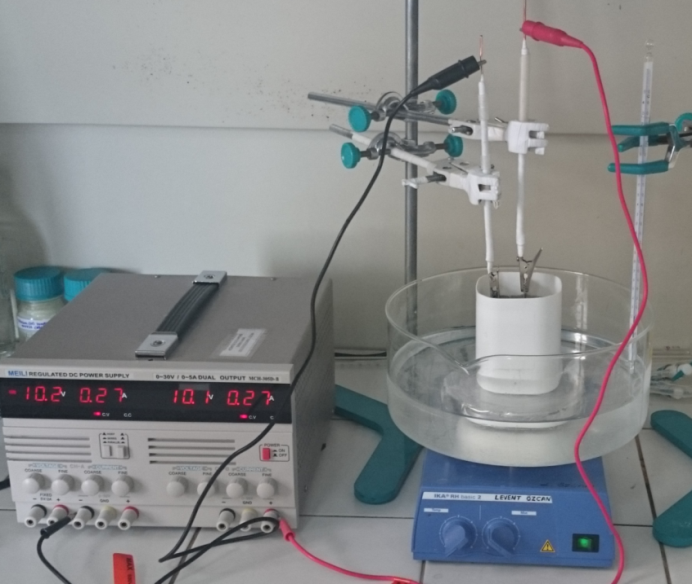


**Figure S1.** Two-electrode system in which the anodic oxidation process is applied to form nanostructured TiO_2_s on the Ti plate surface.


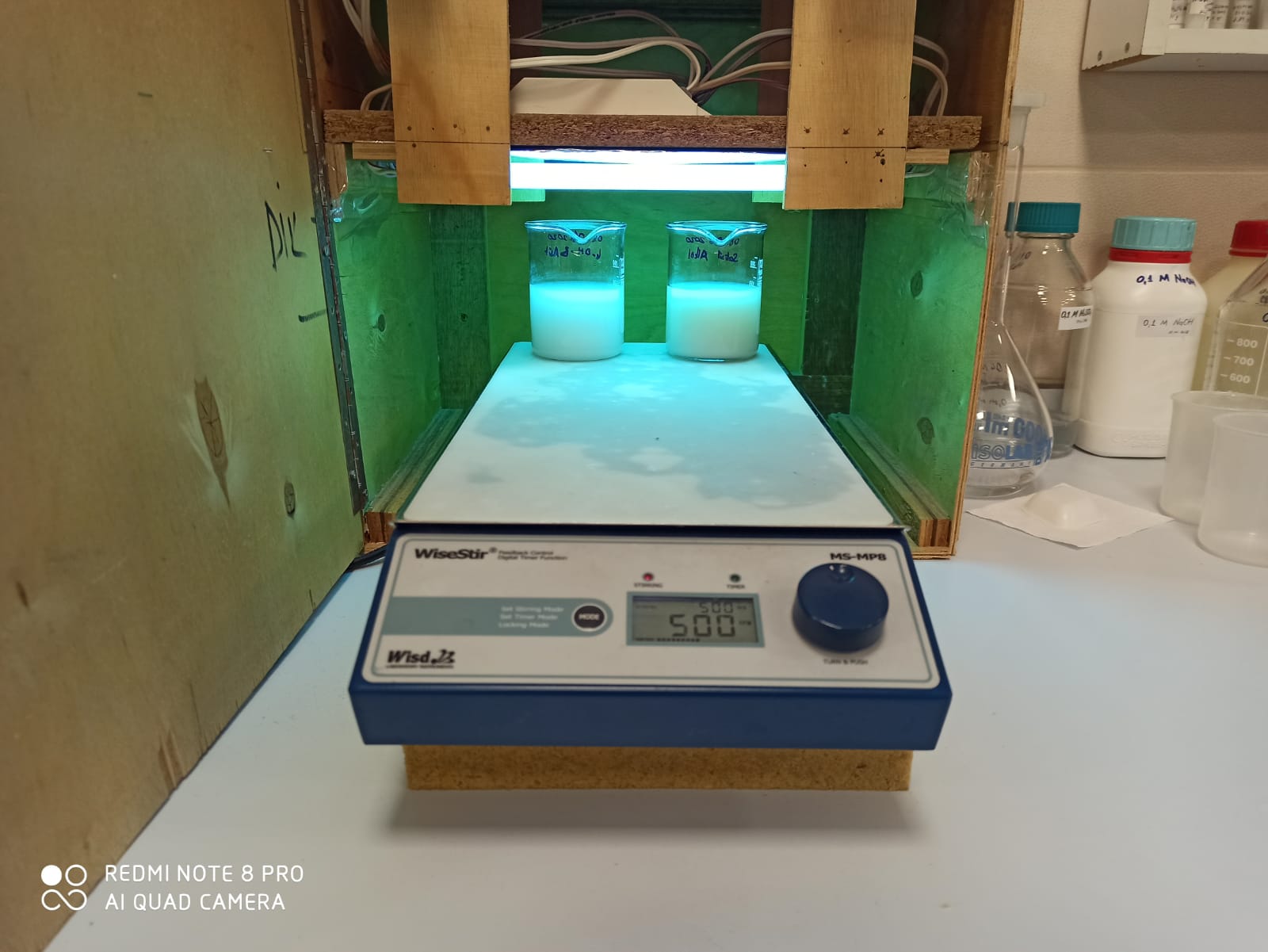

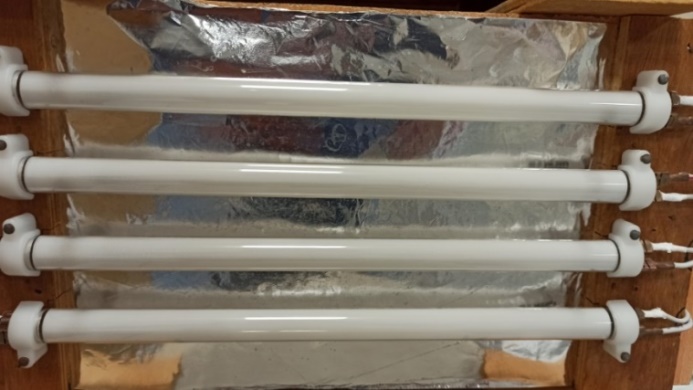


**Figure S2.** Photocatalytic test system and pictures of lamps used in this system.

**Figure S3.** Spectrum of a UVA fluorescent lamp (Philips, 8W).

**Figure S4.** XRD patterns of Merck and Degussa P25 photocatalysts.

**
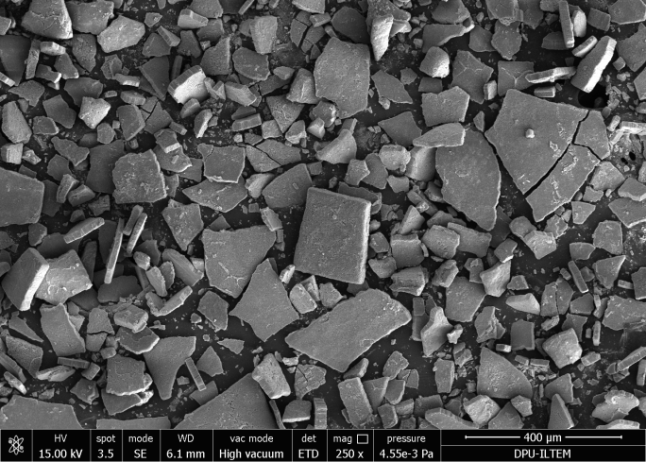

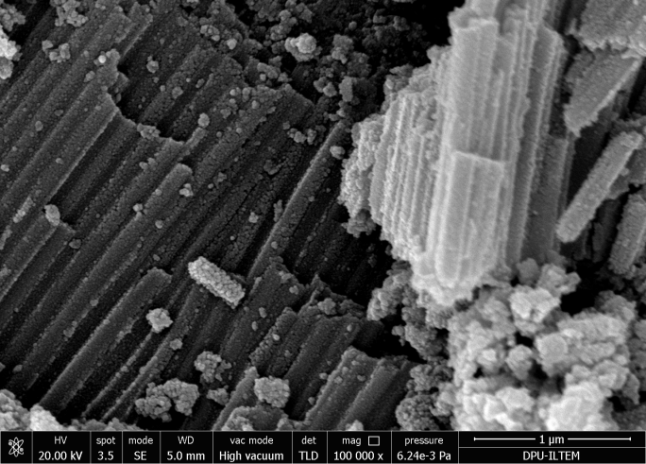
**

**(a) (b)**

**
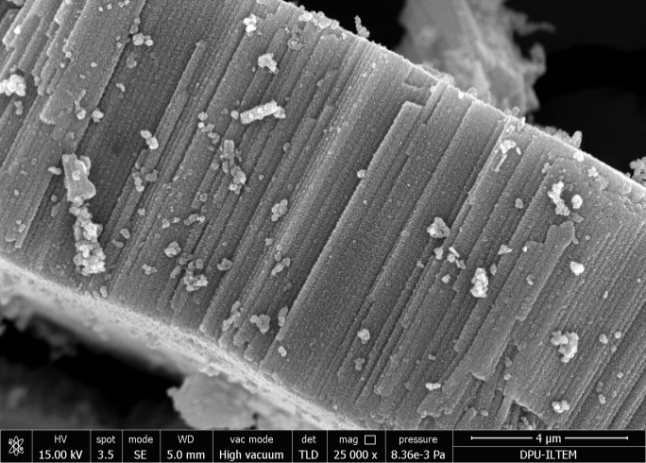

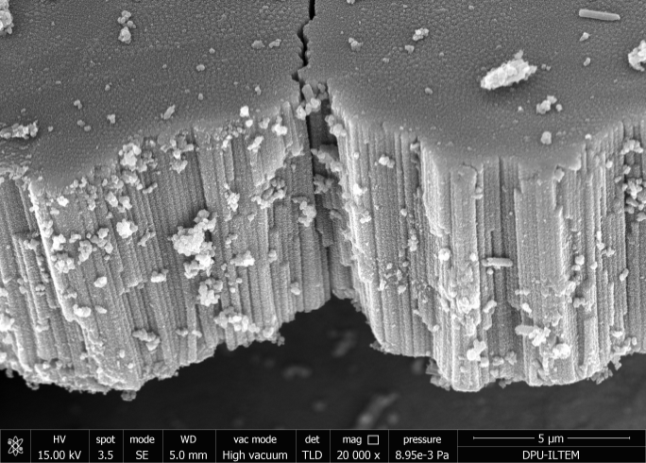
**

**(c) (d)**

**Figure S5.** SEM image of TiO_2_-60V-5h-25 catalyst.

**
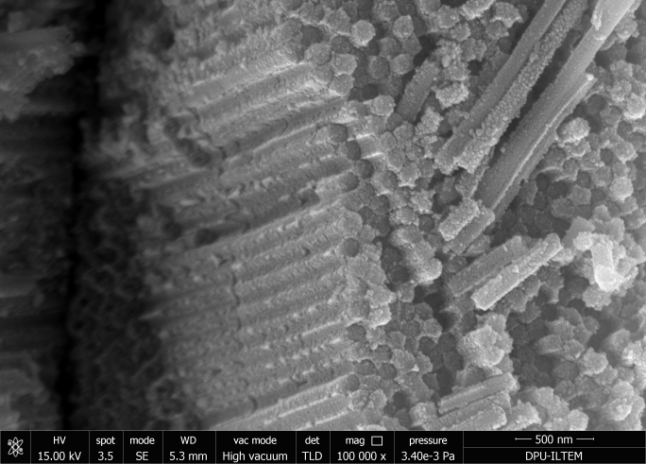

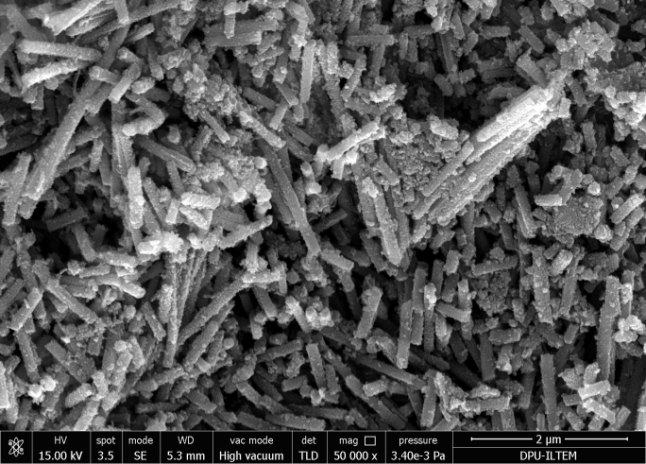
**

**(a) (b)**

**
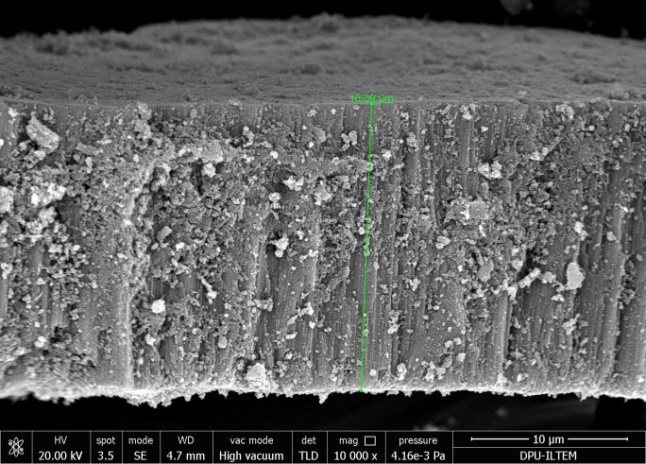
**

**(c)**

**Figure S6.** SEM image of TiO_2_-60V-5h-250 catalyst.

**
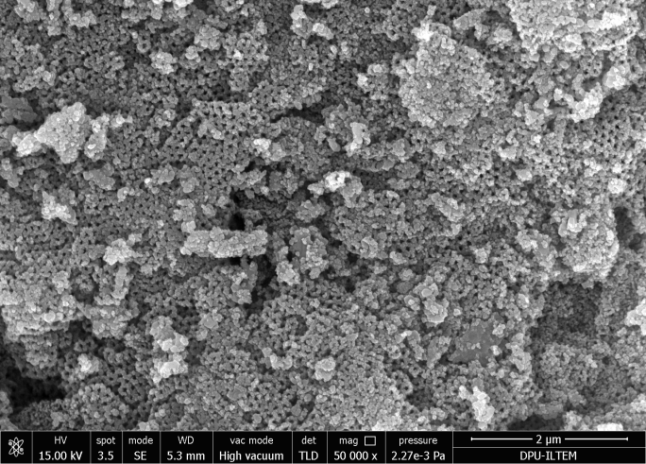

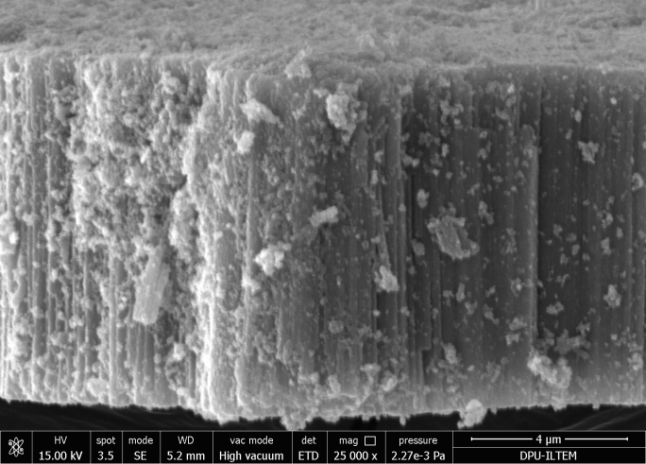
**

**(a) (b)**

**Figure S7.** SEM image of TiO_2_-60V-5h-700 catalyst.

**
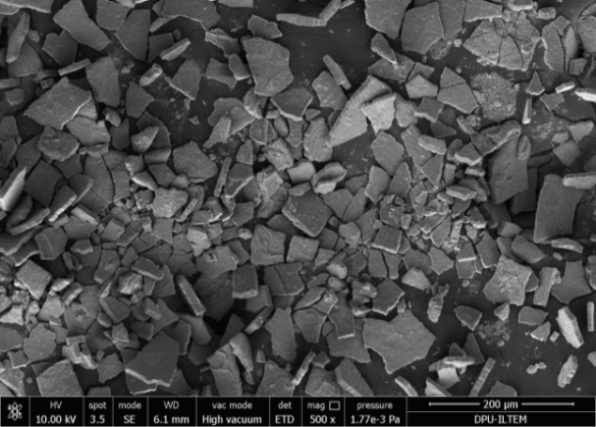

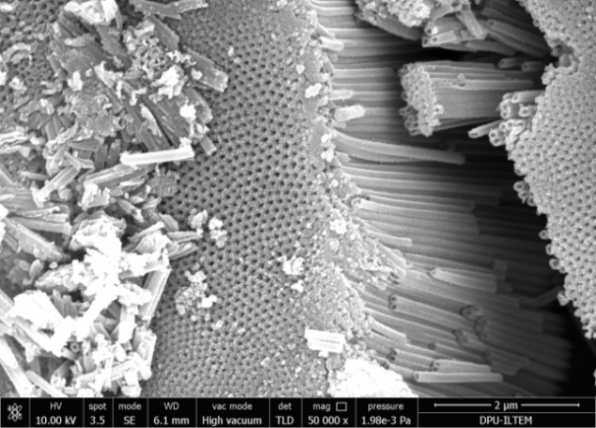
**

**(a) (b)**

**
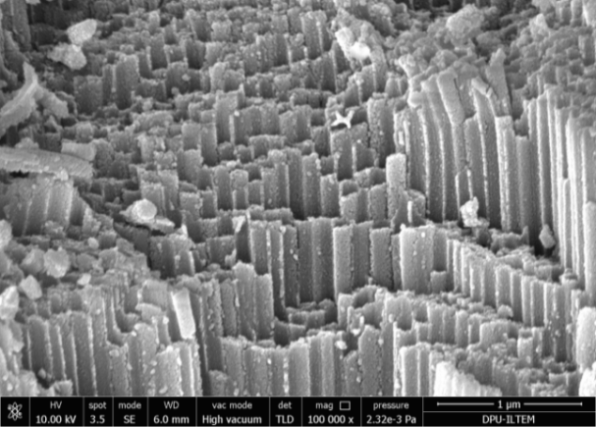

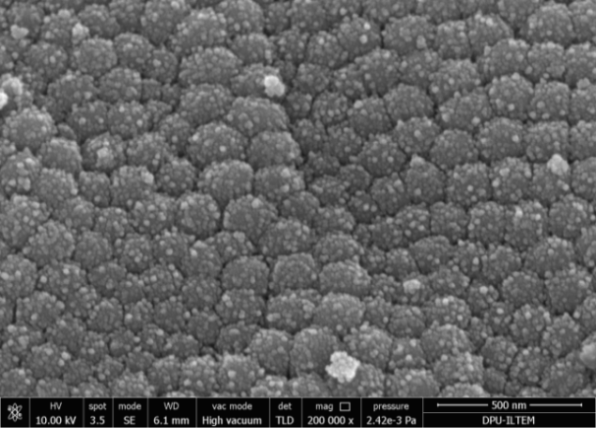
**

**(c) (d)**

**
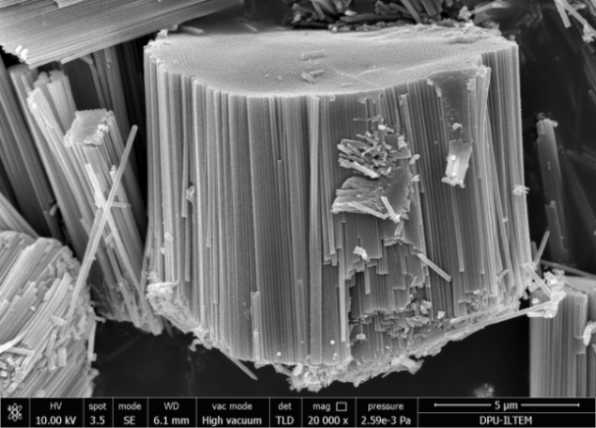
**

**(e)**

**Figure S8.** SEM image of TiO_2_-50V-3h-25 catalyst.

**
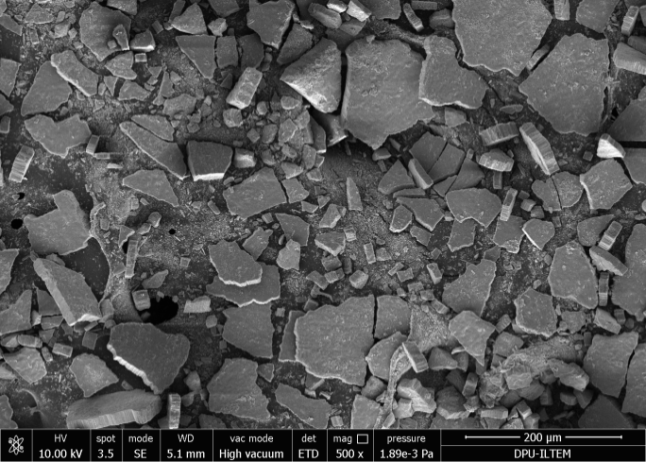

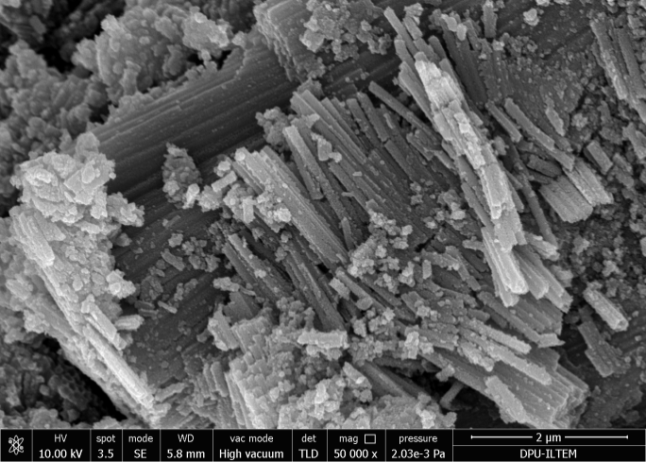
**

**(a) (b)**

**
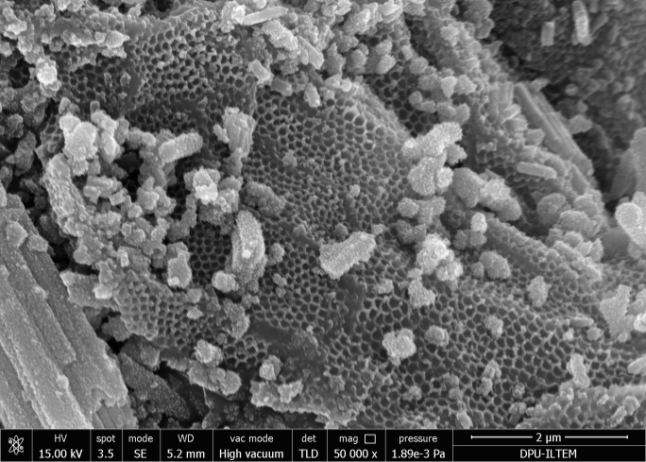

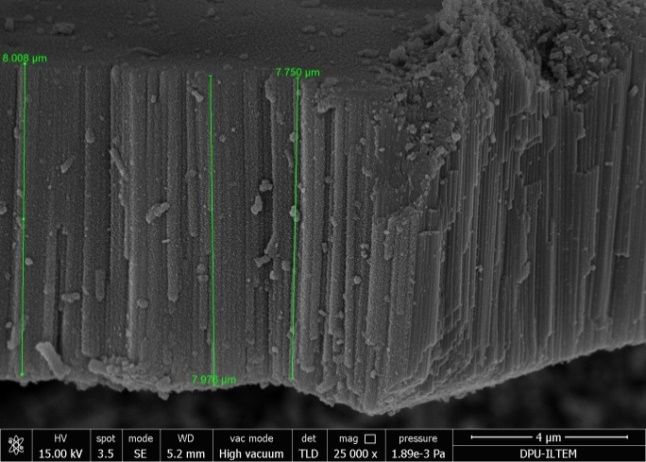
**

**(c) (d)**

**
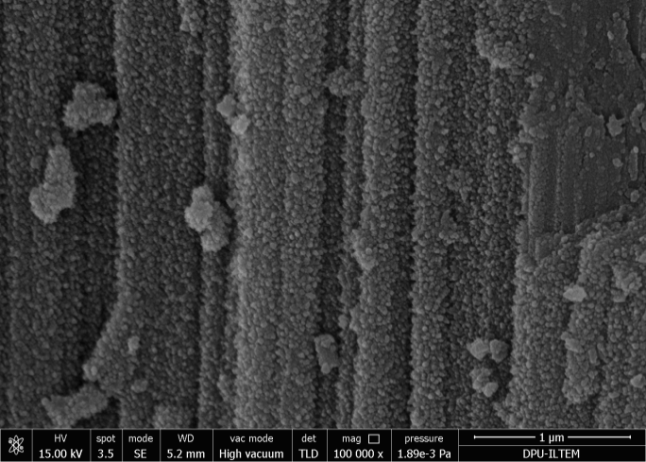

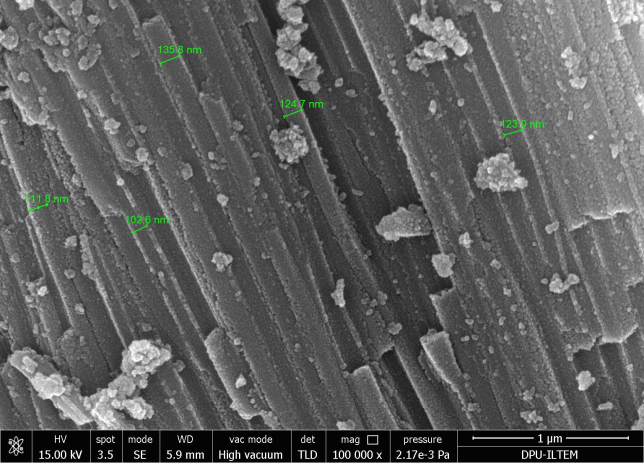
**

**(e) (f)**

**Figure S9.** SEM image of TiO_2_-60V-3h-25 catalyst.

**
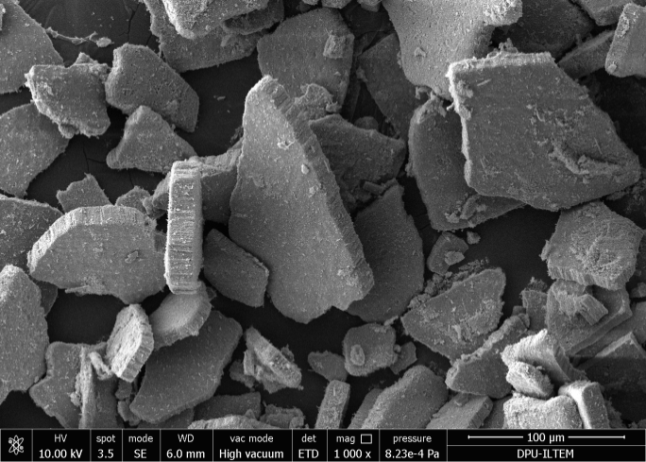

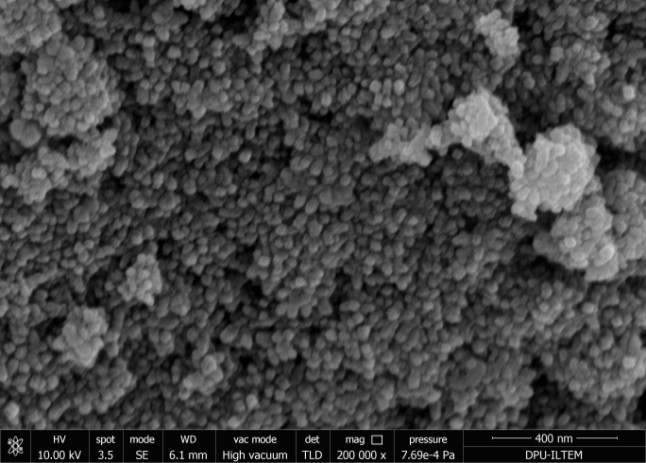
**

**(a) (b)**

**
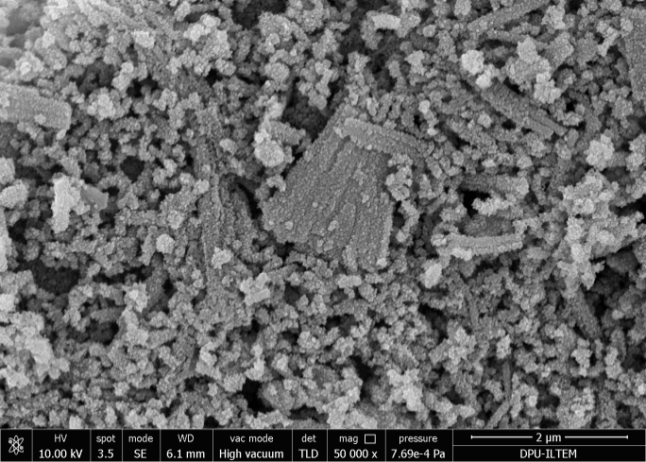

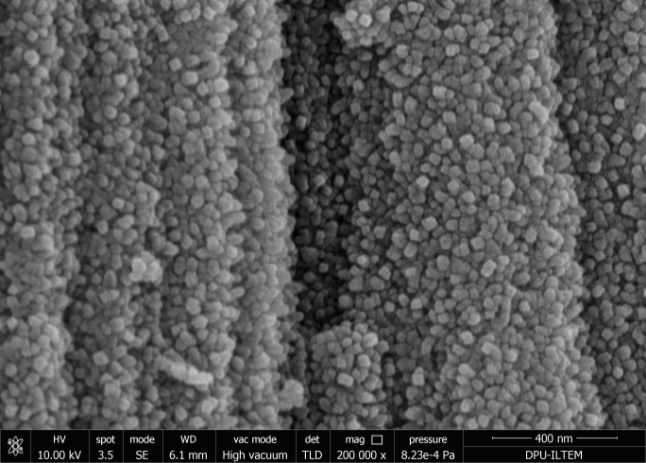
**

**(c) (d)**

**
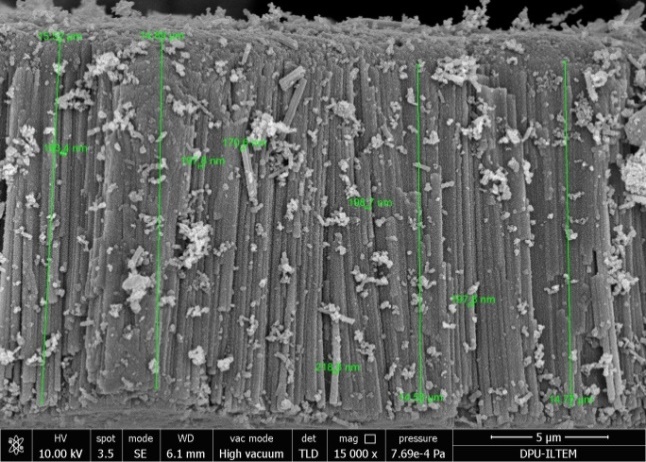

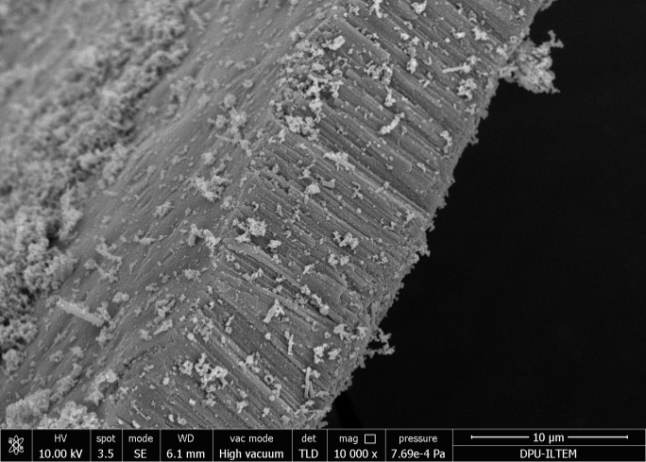
**

**(e) (f)**

**Figure S10.** SEM image of TiO_2_-60V-3h-500 catalyst.
